# Supplementary figures and images for: LINE-1 retrotransposition is a recurrent cause of MET exon 14 skipping in cancer
Source: bioRxiv. 2026 Feb 20:2026.02.19.706876. Preprint. [Version 1] doi: 10.64898/2026.02.19.706876 (PMC12934576; doi:10.64898/2026.02.19.706876)

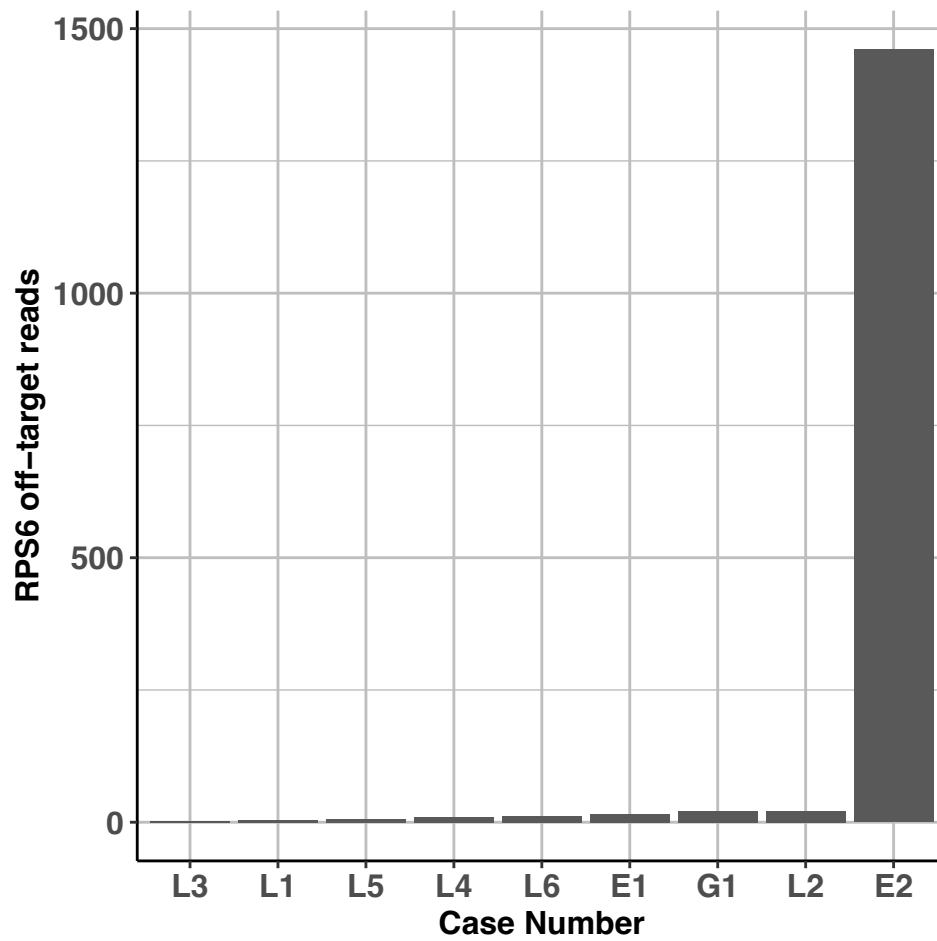

**Figure S1. Number of reads mapping to *RPS6* per case for samples with LINE-1-mediated insertions.**

Supplement: Supplement 2 [file NIHPP2026.02.19.706876v1-supplement-2.pdf]
